# Necroptosis-Related Prognostic Signature and Nomogram Model for Predicting the Overall Survival of Patients with Lung Cancer

**DOI:** 10.1155/2022/4908608

**Published:** 2022-08-31

**Authors:** Yunpeng Xuan, Xiangfeng Jin, Mingzhao Wang, Zizong Wang

**Affiliations:** Department of Thoracic Surgery, The Affiliated Hospital of Qingdao University, Qingdao 266000, Shandong, China

## Abstract

**Background:**

Necroptosis is a type of programmed cell death mode and it serves an important role in the tumorigenesis and tumor metastasis. The purpose of this study is to develop a prognostic model based on necroptosis-related genes and nomogram for predicting the overall survival of patients with lung cancer.

**Method:**

Differentially expressed necroptosis-related genes (NRDs) between lung cancer and normal samples were identified. Univariate and LASSO regression analyses were performed to establish a risk score (RS) model, followed by validation within TCGA and GSE37745. The correlation between RS model and tumor microenvironment, mutation status, or drug susceptibility was analyzed. By combining clinical factors, nomogram was developed to predict 1-, 3-, and 5-year survival probability of an individual. The biological function involved by different risk groups was conducted by GSEA.

**Results:**

A RS model containing six NRDs (*FLNC*, *PLK1*, *ID1*, *MYO1C*, *SERTAD1*, and *LEF1*) was constructed, and patients were divieded into low-risk (LR) and high-risk (HR) groups. Patients in HR group were associated with shorter survival time than those in the LR group; this model had better prognostic performance. Nomogram based on necroptosis score, T stage, and stage had been confirmed to predict survival of patients. The number of resting NK cells and M0 macrophages was higher in HR group. In addition, higher tumor mutational burden and drug sensitivity were observed in the HR group. Patients in HR group were involved in p53 signaling pathway and cell cycle.

**Conclusion:**

This study constructed a robust six-NRDs signature and established a prognostic nomogram for survival prediction of lung cancer.

## 1. Introduction

Lung cancer is a common cancer worldwide and a leading cause of cancer-related death. It is estimated that there are about 1.79 million deaths and 2.2 million new cases each year [1]. With a general understanding of the molecular biology of disease, the application of predictive biomarkers and improvement of therapy methods have positively affected the outcomes of many patients with lung cancer [2]. Meanwhile, the discovery of predicted signatures has created novel opportunities for targeted therapy and immunotherapy of lung cancer [3]. However, effective targets that can be used to predict prognosis and improve clinical treatment of patients are still lacking.

Necroptosis is a type of programmed cell death mode independent of caspase activation, and it is mainly activated by the formation of necrosome [4]. Evidence suggests that necroptosis serves an important role in the biological processes of various tumors, including tumorigenesis and tumor metastasis [5, 6]. However, the regulation mechanism of necroptosis on tumor cells is complex. Some studies suggest that necroptosis cells and cancer are friends. The cell rupture caused by necroptosis releases intracellular components such as inflammatory molecules to the surrounding environment, promoting the inflammatory response, which in turn causes damage to healthy tissue. These damages may ultimately prevent the effectiveness of cancer therapy; in addition, it may promote the spread of cancer cell [7, 8]. Notably, necroptosis is more reported as a foe of cancer. In brief, it may trigger the antitumor immunity in cancer treatment and can be regarded as a promising new target for tumor therapy [9]. An *in vivo* experimental study shows that induction of necroptosis can significantly increase survival time and reduce the growth and metastasis of tumor in mice with pancreatic cancer [10]. Moreover, the combined treatment of berberine and cisplatin can induce the necroptosis of ovarian cancer cells via activating the RIPK3-MLKL pathway and finally may improve the anticancer effect of chemotherapy drugs [11]. Therefore, it is necessary to discover potential biomarkers related to necroptosis, which can help to improve the early diagnosis and anticancer treatment.

In lung cancer, multiple signal pathways have been reported involving in necroptosis process, such as TNF-alpha pathway [12] and AMPK/mTOR and JNK pathways [13]. A previous study has explored the relationship of necroptosis-related regulators with clinical features and prognosis of patients with lung cancer. For example, Park et al. [14] identified three key regulators related to necroptosis, *RIPK1*, *RIPK3*, and *MLKL*, which were downregulated in nonsmall cell lung cancer (NSCLC); they could be used to predict early recurrence after radical resection. Lim et al. [15] indicated that necroptosis-related factors such as *RIPK3* and *PEL11* were positively associated with survival time of patients with stage I lung squamous cell carcinoma. However, only a few studies have investigated the role of necroptosis-related signatures in the prognosis of lung cancer.

In the present study, we first identified necroptosis-related differentially expressed genes (NRDs) between lung cancer and normal samples and then the construction of a RS model via LASSO regression analysis. Moreover, a nomogram model was generated by a combination with clinical features of patients for predicting the probability of survival in 1, 3, and 5 years. The new developed prognostic model can effectively predict prognosis of lung cancer in clinical practice and can help clinicians to formulate better adjuvant treatment strategies.

## 2. Data Sources and Methods

### 2.1. Data Collection and Preprocessing

The gene expression data and clinical follow-up data of patients with lung cancer were extracted from the Cancer Genome Atlas (TCGA) based on the UCSC Xena platform (https://xenabrowser.net/). After excluding the patients with overall survival (OS) time <30 days, 968 lung cancer and 110 normal samples were remained for further analyses. Meanwhile, gene expression data and corresponding clinical information of GSE37745 were retrieved from the Gene Expression Omnibus (GEO) database (https://www.ncbi.nlm.nih.gov/). This dataset was analyzed on the GPL570 [HG-U133_Plus_2] Affymetrix Human Genome U133 Plus 2.0 Array platform. After removing patients with an OS time of less than 30 days, 194 patients were remained for validation analysis. In addition, necroptosis-related genes were acquired from GeneCard online website (https://www.genecards.org/).

### 2.2. Screening of NRDs between Lung Cancer and Normal Samples

According to the data in TCGA, limma package (version 3.34.7) in R4.1.2 was applied to compare the tumor and normal samples. Differentially expressed genes (DEGs) in lung cancer *vs*. normal were identified with the thresholds of false discovery rate (FDR) < 0.05 and |log_2_fold change (FC)| > 1. Pheatmap (version 1.0.8) was used to plot a heatmap for displaying the DEGs. Then, Venn analysis was performed to integrate the DEGs and necroptosis-related genes, and the overlapping genes were defined as NRDs.

### 2.3. Identification of Prognosis-Related NRDs

To assess the prognostic value of NRDs, univariate Cox regression analysis in survival package (version 3.2–13) was performed. Genes with *p* < 0.05 were regarded as prognosis-related NRDs and selected for further analysis.

### 2.4. Construction and Verification of RS Model

The prognosis-related NRDs were entered into the LASSO algorithm using lars package (version 1.2), and optimal gene composition was screened via penalty parameter tuning conducted by 10-fold cross-validation. Then, stepwise Cox regression analysis was performed to build RS model. Necroptosis score (NS) was calculated using the formula: h0 (t)*∗* exp (*β*1X1 + *β*2X2 + ... + *β*nXn); *β* indicates the regression coefficient and h0(t) indicates the benchmark risk function.

NS for samples in the TCGA and GSE37745 was calculated, and then patients in the two datasets were separately divided into low-risk (LR) and high-risk (HR) groups based on median value of NS. Kaplan-Meier (KM) curve analysis was performed to assess the differences in survival time between LR and HR groups. The predictive performance of this model was assessed using the areas under the curve (AUC) values of the receiver-operating characteristic (ROC) curve.

### 2.5. Correlation Analysis between NS and Clinicopathology Factors

After collecting clinical information of patients with lung cancer in TCGA, the relationship between NS and clinical factors (T, N, and M status and stage) was analyzed.

### 2.6. Correlation Analysis between Different Risk Groups and Tumor Microenvironment (TME)

TME is composed of a variety of immune cells, stromal cells, and extracellular matrix molecules, which can define the immunophenotype of cancer and thus affect the prognosis of patients with cancer [16]. In this study, the proportion of 22 immune cells in patients from TCGA was calculated by using CIBERSORTA (https://cibersort.stanford.edu/index.php). Next, differential immune cells (DICs) between LR and HR groups were screened by using Wilcoxon test.

### 2.7. Mutational Characteristics Analysis of RS Model

Based on the mutational data obtained from TCGA, the mutation of each gene in all samples was counted. The number of mutations was arranged from large to small, and the top 20 mutations were selected for display. Then, the mutation frequency of top 20 genes was calculated by using maftools package (version 2.8.0). Moreover, tumor mutation burden (TMB) of samples was calculated, and differences in TMB between HR and LR groups were compared.

### 2.8. Drug Sensitivity Analysis

The IC50 value of six common drugs (cisplatin, cyclopamine, docetaxel, doxorubicin, gemcitabine, and vinblastine) was collected based on the Genomics of Drug Sensitivity in Cancer (GDSC) database, and then the pRRophetic R package was used to predict the differences in IC50 of patients with lung cancer between LR and HR groups, which could reflect the chemotherapeutic response for each sample. Wilcoxon test was used to assess the difference between the two groups and *p* < 0.05 was regarded as statistically significant.

### 2.9. Independent Prognostic Analysis of RS Model

To assess whether NS and clinical factors were independently prognostic indicators, univariate and multivariate Cox regression analyses were conducted. Factors with *p* < 0.05 were regarded as significant difference. Results were displayed using the forest plot.

### 2.10. Development and Validation of a Nomogram Model

According to the independent prognostic factors, rms package (version 6.2–0) was applied to establish a nomogram model. Calibration curves for the 1, 3, and 5 years were plotted to observe the consistency between predicted and actual prognosis. In addition, the significance of predicting prognosis was evaluated using AUC and KM analysis.

### 2.11. Gene Set Enrichment Analysis (GSEA)

GSEA is an algorithm used to assess whether a gene set shows a statistically significant difference between two biological states [17]. Thus, we used GSEA to investigate the pathways significantly enriched by LR group or HR group; *p* < 0.05 and |NES| > 1 was the cut-off threshold.

### 2.12. Statistical Analysis

All statistical analyses were performed using SPSS version 23.0 and R software version 4.2.0. The R packages “lars” (version 1.2) and “survminer” (version 0.4.9) were used to construct the prognostic model. Meanwhile, the “rms package” (version 6.2–0) was used to develop a nomogram model. KM survival analysis was applied to assess distinctions in prognosis between HR and LR with a log-rank *p* value. The Wilcoxon test was used to evaluate the differences in immune cells or IC50 for drugs between LR and HR groups. A *p* value < 0.05 or FDR <0.05 was considered statistically significant.

## 3. Results

### 3.1. Screening of DEGs

**Figure 1:**
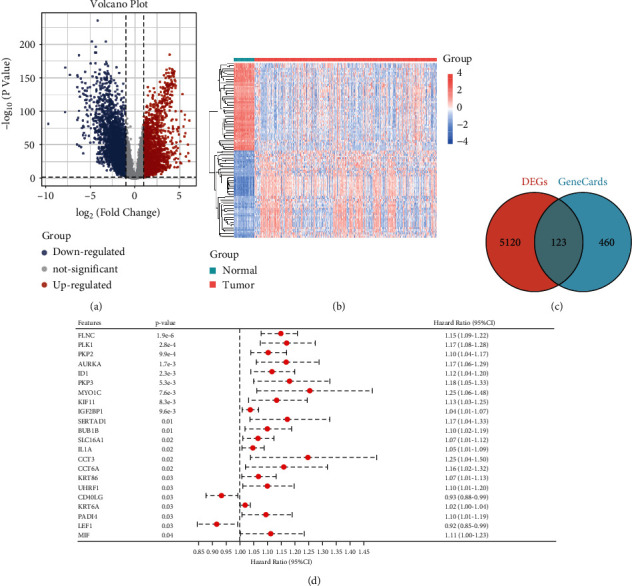
Identification of necroptosis-related differentially expressed genes (NRDs) between lung cancer and normal groups. (a) Volcano plot of differentially expressed genes (DEGs). Blue and red nodes represent down- and upregulated DEGs. (b) Heatmap of top 100 up- and downregulated DEGs. (c) Venn of DEGs and necroptosis-related genes (from GeneCards). (d) Forest plot of prognostic-related NRDs.

After differential expression analysis, 5243 (2173 upregulated and 3070 downregulated) DEGs in lung cancer *vs*. normal were obtained. These DEGs were shown in the volcano plot (Figure 1(a)). Heatmap showed the top 50 up- and downregulated DEGs (total 100, Figure 1(b)), which can clearly divide the samples into two groups (normal and tumor).

Moreover, 583 necroptosis-related genes were collected from GeneCard and then were integrated with DEGs. Finally, 123 NRDs were selected for further analysis (Figure 1(c)).

### 3.2. Screening of Prognosis-Related NRDs

Survival package was used to identify prognosis-related NRDs based on the 123 NRDs. Then, 22 NRDs with prognostic value were obtained (Figure 1(d)).

### 3.3. Generation and Validation of the Six-Gene RS Model

**Figure 2:**
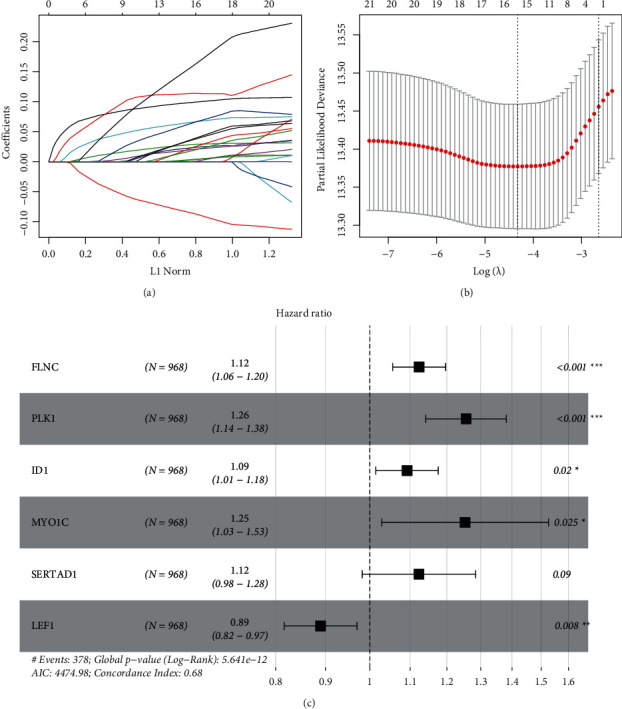
LASSO analysis of 123 prognostic-related NRDs. (a) LASSO coefficient profiles of prognostic-related NRDs. (b) Cross-validation in the LASSO-Cox regression model. (c) Forest plot of six genes obtained from multivariate Cox regression analysis.

LASSO was used to screen optimized gene combination. LASSO coefficient and log*λ* are shown in Figures 2(a) and 2(b). Finally, six prognosis-related NRDs were selected for generation of RS model, including *FLNC*, *PLK1*, *ID1*, *MYO1C*, *SERTAD1*, and *LEF1* (Figure 2(c)). NS was calculated using the formula: RS = 0.099 *∗FLNC* + 0.114 *∗PLK1* + 0.066 *∗ID1* + 0.161 *∗MYO1C* + 0.065 *∗SERTAD1*−0.087 *∗LEF1*.

**Figure 3:**
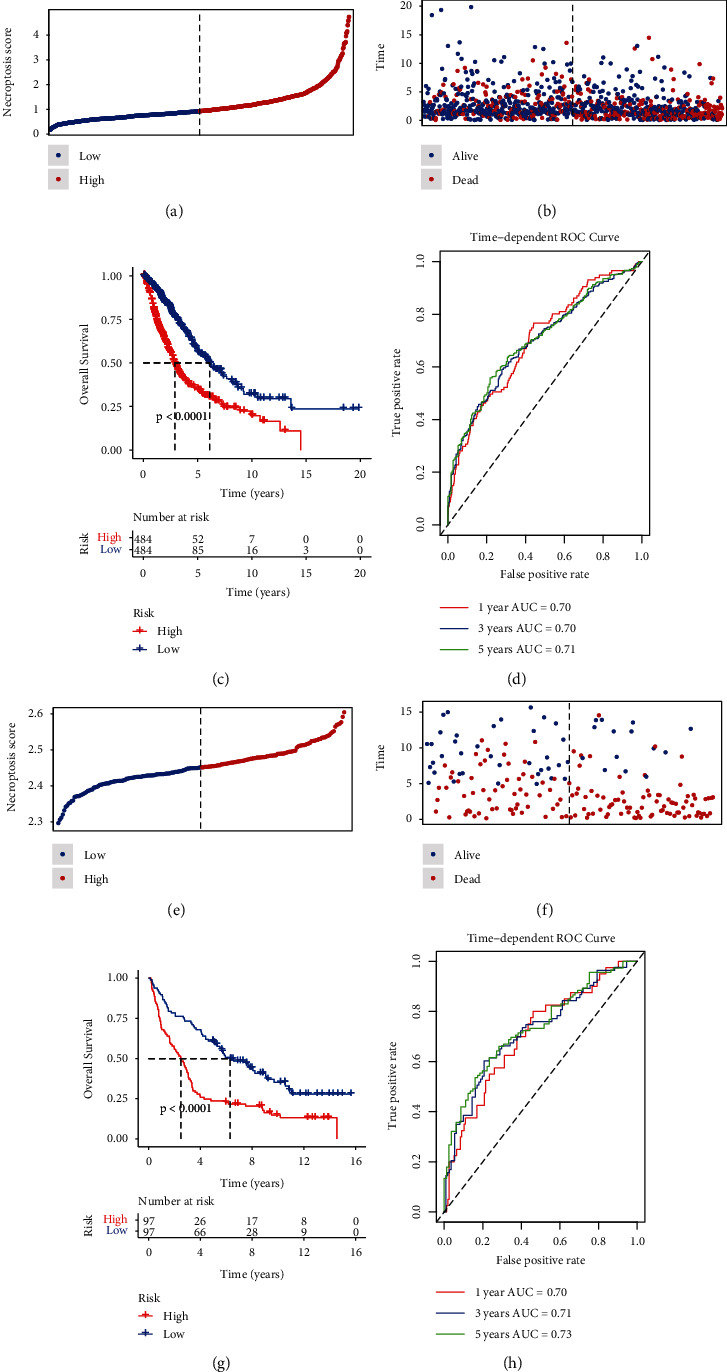
Assessment of prediction performance for RS model in the TCGA and GSE37745 datasets. (a) Distribution of RS in the TCGA set. (b) Distribution of survival status in the TCGA set. (c) KM curve of OS in the TCGA dataset. (d) Time-dependent ROC curve validation at 1-, 3-, and 5-year survival of prognostic rate in the TCGA. (e) Distribution of RS in the GSE37745. (f) Distribution of survival status in the GSE37745. (g) KM curve of OS in the GSE37745. (h) Time-dependent ROC curve for 1, 3, and 5 years survival in the GSE37745.

Next, we verified the predictive performance of the RS model in TCGA and GSE37745 datasets. After calculation of NS, all patients in the two datasets were assigned into LR and HR groups, respectively. As for TCGA data, the distribution of RS for each patient is shown in Figure 3(a); meanwhile, a positive correlation between RS and death cases was observed (Figure 3(b)). Patients in the LR group had significantly improved OS than those in the HR group (*p* < 0.0001, Figure 3(c)). ROC curves showed that the AUC values for 1, 3, 5 years were 0.70, 0.70, and 0.71, respectively (Figure 3(d)). In terms of GSE37745 dataset, more death events were observed in the HR group (Figures 3(e) and 3(f)). Similarly, a significantly longer survival time was found in the LR group than those in the HR group (*p* < 0.0001, Figure 3(g)). AUCs were 0.70, 0.71, and 0.73 achieved in the 1-, 3-, and 5-year survival, respectively (Figure 3(h)). These results indicated that RS model had high reliability and accuracy in predicting the prognosis of patients with lung cancer.

### 3.4. Correlation of NS and Clinical Factors

**Figure 4:**
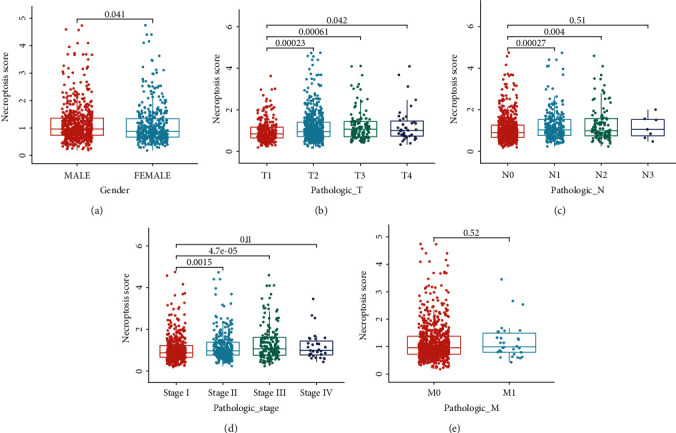
Correlation between clinical characteristics and NS. (a) Gender. (b) T stages. (c) N stages. (d) Stages. (e) M stages.

We also investigated the relationship between NS and clinical factors. Results showed that higher NS was significantly observed in male (*p*=0.041, Figure 4(a)), patients with T3 stage (*p* < 0.001, Figure 4(b)), patients with N1 stage (*p* < 0.001, Figure 4(c)), and patients with stage III (*p* < 0.001, Figure 4(d)). However, no significant correlation was found between NS and M stage (Figure 4(e)).

### 3.5. Correlation of Different Risk Groups with TME

**Figure 5:**
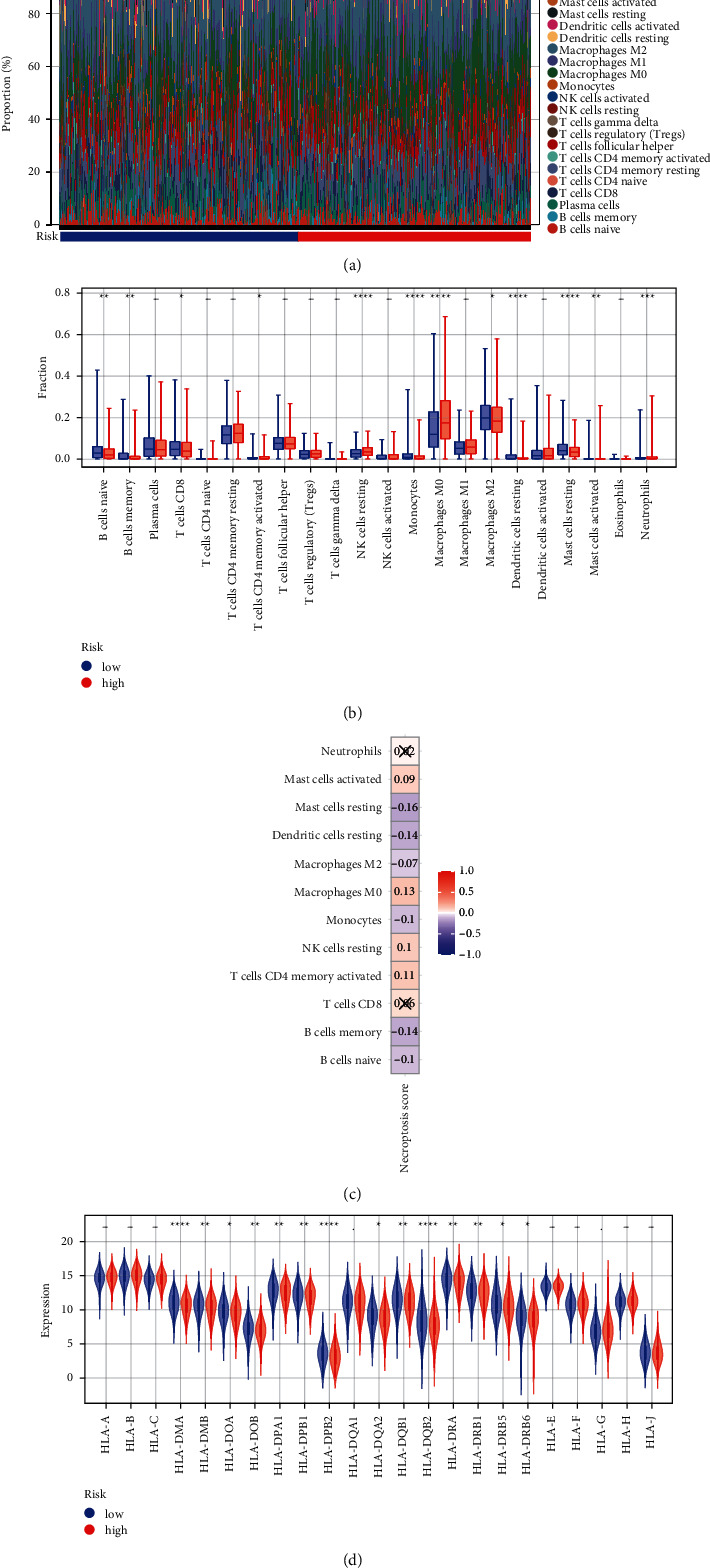
Correlation between different risk groups and tumor microenvironment (TME). (a) Landscape of immune infiltration. (b) Differences in the abundance of immune cells between the HR and LR groups. (c) Relationship between differential immune cells (DICs) and NS. (d) Differences in the expression level of HLA families between the HR and LR groups. ^*∗*^*p* < 0.05, ^*∗∗*^*p* < 0.01, ^*∗∗∗*^*p* < 0.001, and ^*∗∗∗∗*^*p* < 0.0001.

Based on the TCGA data, the landscape of immune infiltration is shown in Figure 5(a). A total of 12 DICs was screened between the LR and HR groups (Figure 5(b)). In brief, the number of resting NK cells and M0 macrophages was higher in the HR group than in the LR group, whereas the number of monocytes and resting mast cells was higher in the LR group. We also assessed the association between 12 DICs and NS. Results revealed that resting mast cells and resting dendritic cells were negatively correlated with RS; M0 macrophages and activated memory CD4 T cells were positively associated with RS (Figure 5(c)).

Differences in the expression level of human leukocyte antigen (HLA) family between the LR and HR groups were also analyzed. Compared with the HR group, the expression levels of several genes, such as *HLA-DMA*, *HLA-DPB2*, *HLA-DQB2*, were significantly increased in the LR group (Figure 5(d)).

### 3.6. Mutation Status of Different Risk Groups

**Figure 6:**
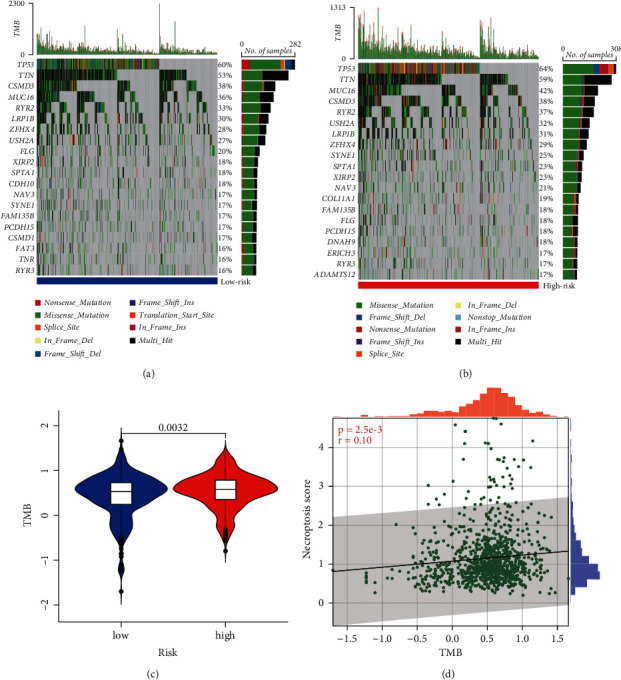
Mutation status of different risk groups. (a) Top 20 mutated genes in the LR group. (b) Top 20 mutated genes in the HR group. (c) Difference in TMB between the LR and HR groups. (d) Relationship between NS and TMB.

Top 20 mutations in patients with lung cancer from the LR and HR groups are displayed in Figures 6(a) and 6(b), respectively. Common mutated genes included *TP53*, *TTN*, *CSMD3*, *MUC16*, *RYR2*, and *LRP18*; the common type of mutation was missense. Meanwhile, significantly higher TMB was observed in the HR group compared to the LR group (*p* = 0.003, Figure 6(c)). A significant positive correlation was found in TMB and NS (*r* = 0.10, *p* = 0.0025, Figure 6(d)).

### 3.7. Sensitivity of Different Risk Groups to Six Chemotherapy Drugs

**Figure 7:**
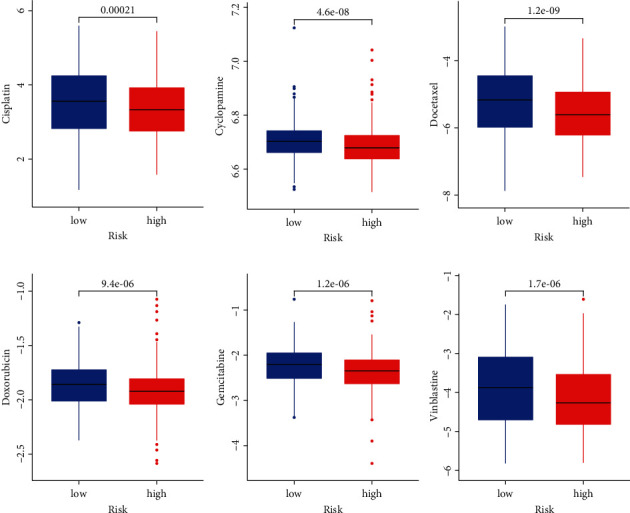
Difference in the IC50 level of six drugs between the LR and HR groups.

Further, the IC50 values of six common chemotherapeutic drugs between the LR and HR groups were compared. Results showed that the IC50 value of six drugs (cisplatin, cyclopamine, docetaxel, doxorubicin, gemcitabine, and vinblastine) was significantly lower in the HR groups than in the LR groups (all *p* < 0.05, Figure 7), indicating that patients in the HR group were more likely to benefit from these agents.

### 3.8. Generation and Validation of a Nomogram Model

**Figure 8:**
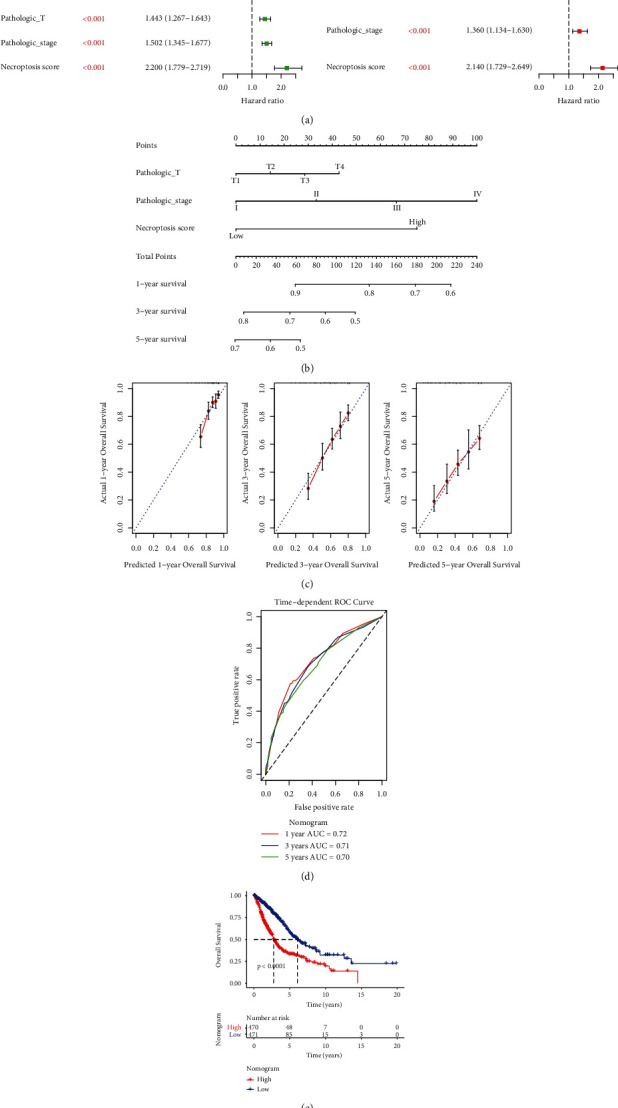
Development and validation of a nomogram model. (a) Univariate and multivariate Cox regression analysis. (b) Nomogram model. (c) Calibration curve of nomogram to observe the predicted and actual 1-, 3-, and 5-year survival. (d) ROC analysis of 1-, 3-, and 5-year survival based on nomogram model. (e) KM curve of the LR and HR groups based on nomogram.

After univariate and multivariate Cox regression analysis, three independent prognostic factors, including T stage, stage, and NS, were screened (*p* < 0.05, Figure 8(a)), which were then used for generation of a nomogram model (Figure 8(b)). In performance evaluation analysis, predicted 1-, 3-, and 5-year survival were similar to the actual survival (Figure 8(c)). Moreover, the AUC values for 1, 3 and 5 years were 0.72, 0.71, and 0.70 (Figure 8(d)). Survival analysis revealed that patients in the LR group had better prognosis based on the nomogram model (Figure 8(e)). Therefore, this nomogram model was a stable and independent prognostic factor for lung cancer.

### 3.9. Functional Pathways of the Different Risk Groups

**Figure 9:**
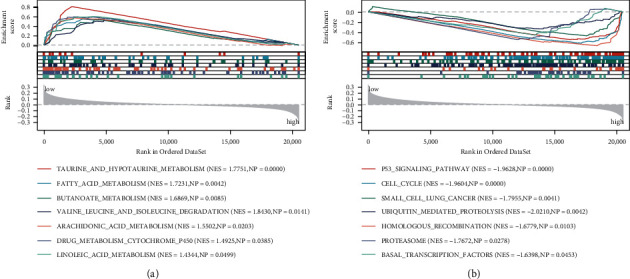
GSEA of the biological function in the LR group (a) and HR group (b).

The potential pathways of the different risk groups were explored by using GSEA, and 14 differential pathways in the LR *vs.* HR groups were obtained. As shown in Figure 9(a), patients in the LR groups were significantly enriched in seven pathways, such as taurine and hypotaurine metabolism, fatty acid metabolism, and butanoate metabolism. Meanwhile, patients in the HR group were also involved in seven pathways, such as p53 signaling pathway, cell cycle, small cell lung cancer, and ubiquitin mediated proteolysis (Figure 9(b)).

## 4. Discussion

Necroptosis is involved in the pathogenesis of many diseases, such as cardiovascular disease, cancer, and metabolic disease [18]. Increasing research has indicated that necroptosis plays an important role in tumor cells. Several therapeutic drugs have developed to work against cancer via manipulating necroptosis [19]. Moreover, necroptosis regulators may be regarded as biomarkers for prognosis of cancer [20]. In this study, NRDs between lung cancer and normal samples were identified, and then the correlation between NRDs and clinical outcomes of patients with lung cancer was assessed. Finally, a RS model consisting of six NRDs was established, which could independently predict prognosis of patients with lung cancer. Furthermore, we also evaluated the relationship between different risk groups and immune infiltration as well as mutation status, revealing that necroptosis may affect the prognosis of patients with lung cancer through regulating TME and TMB.

A total of six genes were involved in the RS model: *FLNC*, *PLK1*, *ID1*, *MYO1C*, *SERTAD1*, and *LEF1*. Filamin C (*FLNC*) encodes gamma filament protein and is involved in the anchoring of membrane proteins on the actin cytoskeleton [21]. Shi et al. [22] indicated that methylation of *FLNC* was associated with poor prognosis of patients with gastric cancer. Polo-like kinase1 (*PLK1*) is highly expressed during mitosis and is overexpressed in various types of tumors; it has been confirmed as a potential therapeutic target for cancer [23]. Previous study showed that *PLK1* was upregulated in prostate cancer cell, and necroptosis regulated by inhibition of *PLK1* might be an effective intervention for castration-resistant prostate cancer [24]. Inhibitor of DNA binding 1 (*ID1*) is involved in cell differentiation and cell cycle, which may serve a role in the occurrence and metastasis of various tumors [25]. The prognostic value of *ID1* was reported in lung cancer and overexpression of *ID1* was connected with poor survival of patients with lung adenocarcinoma [26]. Tan et al. [27] showed that necroptosis induced by *ID1* overexpression enhanced the sensitivity of NSCLC to gefitinib treatment. Myosin IC (*MYO1C*) was differentially expressed in lung cancer and normal tissues [28]. SERTA domain containing 1 (*SERTAD1*) plays roles in different types of cell death response [29] and is regarded as a key nuclear transcription factor in carcinogenesis, including lung cancer [30]. Lymphoid enhancer binding factor 1 (*LEF1*) belongs to the LEF/T-cell factor family, and mutations in this gene are found in cancers, especially somatic sebaceous tumor [31]. Previous study indicated that knockdown of *LEF1* resulted in TNF-*α* induced necroptosis in chronic lymphocytic leukemia cells [32]. In addition, knockdown of *LEF1* inhibited migration of lung cancer [33]. Taken together, the above studies suggested that these genes had a certain relationship with the necroptosis of cancer as well as prognosis of patients.

Based on this RS model, all patients were assigned to the LR and HR groups. Patients in the HR groups had poor survival; this model with good predictive performance could independently predict the prognosis of patients with lung cancer. Next, a nomogram model containing T stage, stage, and NS was established, which could predict the 1-, 3-, and 5-year survival probabilities of individuals and was consistent with the actual probabilities. Together, the new developed necroptosis-related signature and nomogram were effective indicators for predicting the outcome of patients with lung cancer, and these models could assist clinicians to formulate better strategies for lung cancer treatment.

The correlation between risk groups and immune cells or mutations was also analyzed in this study. Higher fractions of resting NK cells and M0 macrophages were observed in the HR group. Consistent with the study of Cai et al. [34], they also found a higher infiltration of these two immune cells in the HR group of patients with lung adenocarcinoma. NK cells are innately selective for tumor cells and can serve as a promising tool for cancer immunotherapy [35]. Meanwhile, the proportion of NK cells were markedly related to survival time of lung cancer [36]. Increased numbers of M0 macrophages were contributed to poor prognosis of patients with early lung cancer [37], which was also observed in our study. TMB is an effective prognostic predictor in patients with lung cancer, suggesting that the higher level of TMB was associated with shorter survival time [38]. We also observed the same results in this analysis; specifically, patients in the HR group had higher TMB level. In terms of drug response, we discovered that patients in the HR group were linked to higher drug sensitivity for cisplatin, cyclopamine, docetaxel, doxorubicin, gemcitabine, and vinblastine, indicating that patients with LR score might benefit from these drugs' therapy.

Further, the biological function enriched by the LR and HR groups was explored. Several pathways, such as p53 signaling pathway and cell cycle, were observably enriched in the HR group. The findings were also revealed by Jin et al. [39], and these pathways could be regulated by a prognostic factor (namely *RRM2*) for lung cancer. Abnormalities of p53 signaling pathway exist in almost all cancers, and therapeutic methods targeting this pathway have attracted extensive attention [40]. Dysregulation of the cell cycle is a common event in NSCLC and adversely affects prognosis; this maladjustment also leads to uncontrolled cell proliferation and has been observed in lung cancer [41]. Previous research pointed out that the cell cycle offered predictive, prognostic, and therapeutic possibilities for cancer, and further in-depth study of this pathway may improve the status of patients with lung cancer [42].

To our knowledge, this study is the first to establish a risk model based on the necroptosis-related signature and then investigate its prognostic value in lung cancer. However, this analysis still has certain limitations. First, the specific function of the identified genes in lung cancer or necroptosis process has not been investigated. Second, when developing the nomogram model, some clinical factors that may affect the prognostic of patients with lung cancer were not taken into account, such as gender, smoking status, and treatment method. Furthermore, our results were only validated in one dataset. Finally, all patients included in our study were lung cancer, and we did not distinguish the specific subtype of each patient, such as lung adenocarcinoma or lung squamous cell carcinoma. Consequently, we will incorporate more complete clinicopathological factors and detailed treatment information and then perform validation analyses in other datasets to confirm the predictive ability and accuracy of this model.

## 5. Conclusion

A novel prognostic model involving six necroptosis-related genes was constructed to predict the survival time of patients with lung cancer. The model with high sensitivity and specificity may serve as an independent prognostic factor for lung cancer. These six genes may be useful for elucidating the molecular mechanisms related to necroptosis and may be considered as potential therapeutic targets for lung cancer.

## Data Availability

The data could be downloaded at https://xenabrowser.net/, https://www.ncbi.nlm.nih.gov/, and https://www.genecards.org/, and the codes used during the present study are available from the corresponding author on reasonable request.

## Additional Points

(1) A prognostic model involving six necroptosis-related genes was constructed. (2) Genes included in the model were *FLNC, PLK1, ID1, MYO1C, SERTAD1, and LEF1*. (3) A prognostic model and a nomogram had better predictive performance. (4) The number of resting NK cells and M0 macrophages was higher in the HR group.

## References

[1] Thai A. A., Solomon B. J., Sequist L. V., Gainor J. F., Heist R. S. (2021). Lung cancer. *The Lancet*.

[2] Meng L., Xu J., Ye Y., Wang Y., Luo S., Gong X. (2021). The combination of radiotherapy with immunotherapy and potential predictive biomarkers for treatment of non-small cell lung cancer patients. *Frontiers in Immunology*.

[3] Burotto M., Thomas A., Subramaniam D., Giaccone G., Rajan A. (2014). Biomarkers in early-stage non-small-cell lung cancer: current concepts and future directions. *Journal of Thoracic Oncology*.

[4] Zhang Y., Su S. S., Zhao S. (2017). RIP1 autophosphorylation is promoted by mitochondrial ROS and is essential for RIP3 recruitment into necrosome. *Nature Communications*.

[5] Seehawer M., Heinzmann F., D’Artista L. (2018). Necroptosis microenvironment directs lineage commitment in liver cancer. *Nature*.

[6] Yan J., Wan P., Choksi S., Liu Z.-G. (2022). Necroptosis and tumor progression. *Trends in Cancer*.

[7] Berghe T. V., Linkermann A., Jouan-Lanhouet S., Walczak H., Vandenabeele P. (2014). Regulated necrosis: the expanding network of non-apoptotic cell death pathways. *Nature Reviews Molecular Cell Biology*.

[8] Philipp S., Sosna J., Adam D. (2016). Cancer and necroptosis: friend or foe?. *Cellular and Molecular Life Sciences*.

[9] Liu W., Jin W., Zhu S., Chen Y., Liu B. (2022). Targeting regulated cell death (RCD) with small-molecule compounds in cancer therapy: a revisited review of apoptosis, autophagy-dependent cell death and necroptosis. *Drug Discovery Today*.

[10] Xie Y., Zhu S., Zhong M. (2017). Inhibition of aurora kinase A induces necroptosis in pancreatic carcinoma. *Gastroenterology*.

[11] Liu L., Fan J., Ai G. (2019). Berberine in combination with cisplatin induces necroptosis and apoptosis in ovarian cancer cells. *Biological Research*.

[12] Yu W.-N., Lai Y.-J., Ma J.-W. (2019). Citronellol induces necroptosis of human lung cancer cells *via* TNF-*α* pathway and reactive oxygen species accumulation. *In Vivo*.

[13] Ge D., Tao H.-R., Fang L. (2020). 11-Methoxytabersonine induces necroptosis with autophagy through AMPK/mTOR and JNK pathways in human lung cancer cells. *Chemical and Pharmaceutical Bulletin*.

[14] Park J. E., Lee J. H., Lee S. Y. (2020). Expression of key regulatory genes in necroptosis and its effect on the prognosis in non-small cell lung cancer. *Journal of Cancer*.

[15] Lim J. H., Oh S., Kim L. (2021). Low-level expression of necroptosis factors indicates a poor prognosis of the squamous cell carcinoma subtype of non-small-cell lung cancer. *Translational Lung Cancer Research*.

[16] Binnewies M., Roberts E. W., Kersten K. (2018). Understanding the tumor immune microenvironment (TIME) for effective therapy. *Nature Medicine*.

[17] Canzler S., Hackermüller J. (2020). multiGSEA: a GSEA-based pathway enrichment analysis for multi-omics data. *BMC Bioinformatics*.

[18] Liu Y., Liu T., Lei T. (2019). RIP1/RIP3-regulated necroptosis as a target for multifaceted disease therapy (Review). *International Journal of Molecular Medicine*.

[19] Fulda S. (2014). Therapeutic exploitation of necroptosis for cancer therapy. *Seminars in Cell & Developmental Biology*.

[20] Zhao Z., Liu H., Zhou X. (2021). Necroptosis-related lncRNAs: predicting prognosis and the distinction between the cold and hot tumors in gastric cancer. *Journal of Oncology*.

[21] Guyon J. R., Kudryashova E., Potts A. (2003). Calpain 3 cleaves filamin C and regulates its ability to interact with gamma- and delta-sarcoglycans. *Muscle & Nerve*.

[22] Shi J., Zhang G., Yao D. (2012). Prognostic significance of aberrant gene methylation in gastric cancer. *American Journal of Cancer Research*.

[23] Abdelfatah S., Berg A., Huang Q. (2019). MCC1019, a selective inhibitor of the Polo-box domain of Polo-like kinase 1 as novel, potent anticancer candidate. *Acta Pharmaceutica Sinica B*.

[24] Deeraksa A., Pan J., Sha Y. (2013). Plk1 is upregulated in androgen-insensitive prostate cancer cells and its inhibition leads to necroptosis. *Oncogene*.

[25] Zhao Z., Bo Z., Gong W., Guo Y. (2020). Inhibitor of differentiation 1 (Id1) in cancer and cancer therapy. *International Journal of Medical Sciences*.

[26] Antonângelo L., Tuma T., Fabro A. (2016). Id-1, Id-2, and Id-3 co-expression correlates with prognosis in stage I and II lung adenocarcinoma patients treated with surgery and adjuvant chemotherapy. *Experimental Biology and Medicine*.

[27] Tan H.-Y., Wang N., Chan Y.-T. (2020). ID1 overexpression increases gefitinib sensitivity in non-small cell lung cancer by activating RIP3/MLKL-dependent necroptosis. *Cancer Letters*.

[28] Huang L., Chen L., Gui Z. X., Liu S., Wei Z. J., Xu A. M. (2020). Preventable lifestyle and eating habits associated with gastric adenocarcinoma: a case-control study. *Journal of Cancer*.

[29] Jung S., Li C., Duan J. (2015). TRIP-Br1 oncoprotein inhibits autophagy, apoptosis, and necroptosis under nutrient/serum-deprived condition. *Oncotarget*.

[30] Mongre R. K., Jung S., Mishra C. B., Lee B., Kumari S., Lee M. S. (2019). Prognostic and clinicopathological significance of SERTAD1 in various types of cancer risk: a systematic review and retrospective analysis. *Cancers*.

[31] Petersson M., Reuter K., Brylka H., Kraus A., Schettina P., Niemann C. (2015). Interfering with stem cell-specific gatekeeper functions controls tumour initiation and malignant progression of skin tumours. *Nature Communications*.

[32] Wu W., Zhu H., Fu Y. (2016). High LEF1 expression predicts adverse prognosis in chronic lymphocytic leukemia and may be targeted by ethacrynic acid. *Oncotarget*.

[33] Li B., Zhu L., Li L., Ma R. (2021). lncRNA OXCT1-AS1 promotes metastasis in non-small-cell lung cancer by stabilizing LEF1, in vitro and in vivo. *BioMed Research International*.

[34] Cai S., Guo X., Huang C. (2021). Integrative analysis and experiments to explore angiogenesis regulators correlated with poor prognosis, immune infiltration and cancer progression in lung adenocarcinoma. *Journal of Translational Medicine*.

[35] Kwon H.-J., Kim N., Kim H. S. (2017). Molecular checkpoints controlling natural killer cell activation and their modulation for cancer immunotherapy. *Experimental and Molecular Medicine*.

[36] Hu B., Shi X., Du X., Xu M., Wang Q., Zhao H. (2020). Pattern of immune infiltration in lung cancer and its clinical implication. *Clinica Chimica Acta*.

[37] Liu X., Wu S., Yang Y., Zhao M., Zhu G., Hou Z. (2017). The prognostic landscape of tumor-infiltrating immune cell and immunomodulators in lung cancer. *Biomedicine & Pharmacotherapy*.

[38] Hellmann M. D., Nathanson T., Rizvi H. (2018). Genomic features of response to combination immunotherapy in patients with advanced non-small-cell lung cancer. *Cancer Cell*.

[39] Jin C.-Y., Du L., Nuerlan A. H., Wang X. L., Yang Y. W., Guo R. (2020). High expression of RRM2 as an independent predictive factor of poor prognosis in patients with lung adenocarcinoma. *Aging (Albany NY)*.

[40] Huang J. (2021). Current developments of targeting the p53 signaling pathway for cancer treatment. *Pharmacology & Therapeutics*.

[41] Malumbres M., Barbacid M. (2009). Cell cycle, CDKs and cancer: a changing paradigm. *Nature Reviews Cancer*.

[42] Vincenzi B., Schiavon G., Silletta M. (2006). Cell cycle alterations and lung cancer. *Histology & Histopathology*.

